# Self-Esteem, Wellbeing, and Health Status of Female Athletes in the Context of Sport Performance

**DOI:** 10.1089/whr.2024.0119

**Published:** 2025-01-14

**Authors:** Taysir Laajili, Csaba Kató, Zsolt Gábor Szabó, Andrea Lukács

**Affiliations:** ^1^ School of Doctoral Studies, Hungarian University of Sport Sciences, Budapest, Hungary.; ^2^ Faculty of Health Sciences, University of Miskolc, Miskolc, Hungary.

**Keywords:** female athletes, health, individual sport, self-esteem, team sport, wellbeing

## Abstract

**Background:**

Competing at a high level is challenging for athletes, both mentally and physically. Intense sport engagement is not always beneficial for health. This study investigates and compares self-esteem, wellbeing, and health status among athletes at different levels. In addition, it examines whether the type of sport (individual versus team) affects the self-esteem and wellbeing.

**Methods:**

A quantitative, observational survey was conducted, involving 296 female participants aged 18–35 years divided into three groups based on their sport performance: high-performance athletes (41%), recreational athletes (27%), and individuals with no or lower levels of physical activity (32%). The Rosenberg Self-Esteem Scale, WHO-5 Well-Being Index, and the EuroQol Visual Analogue Scale were used to measure self-esteem, wellbeing, and health status, respectively.

**Results:**

High-performance athletes exhibited significantly higher self-esteem, wellbeing, and health status compared with individuals with no or lower levels of physical activity. Recreational athletes showed better wellbeing than individuals with no or lower levels of physical activity and had no significant differences from high-performance athletes in any parameter. Results were not affected by whether the sport was individual or team-based.

**Conclusions:**

These findings highlight that regular sport participation, whether at a recreational or high-performance level, provides substantial mental and physical health benefits, while individuals with no or lower levels of physical activity experience significantly lower levels of self-esteem, subjective wellbeing, and health. The findings of this study suggest that regular sport activity, regardless of competitive level, provides substantial benefits, underscoring the importance of maintaining a physically active lifestyle.

## Introduction

For a high-performance athlete to excel in their sport, it is crucial that they feel good both physically and mentally, and that they possess adequate self-awareness and self-esteem to achieve success. Regular exercise has numerous preventive and positive effects, such as reducing the risk of cardiovascular diseases, improving sleep quality, and enhancing mental health.^1–4^ However, competitive sports present different challenges for the athlete. Coping during training and competition imposes greater mental burdens, which can increase levels of stress and anxiety.^5–8^ Excessive training without adequate rest can lead to overtraining syndrome, characterized by fatigue, decreased performance, and an increased risk of injury.^9,10^ Athletes may sacrifice their health, homes, education, and social development for the sake of sporting “success,” which can have long-term negative effects on their quality of life.^11^ Bartholomew et al. found that when athletes feel their basic psychological needs—such as autonomy, competence, and relatedness—are not being met, it can lead to negative outcomes such as burnout, stress, and poor mental health. Conversely, when these psychological needs are satisfied, it can enhance their wellbeing and performance.^12^

Recreational athletes engage in sports and physical activities primarily for enjoyment, health, and social interaction rather than for professional or competitive reasons. The majority are motivated by the health benefits, including improved cardiovascular health, stronger muscles and bones, better weight management, and enhanced mental wellbeing.^13–15^ While not focused on competition, many recreational athletes set personal goals, such as completing a marathon, improving their personal best times, or mastering a new skill. Achieving these goals can provide a sense of accomplishment and boost self-esteem.

Good self-esteem involves having a positive and balanced view of oneself, recognizing one’s worth, abilities, and inherent value. It includes self-acceptance, confidence, resilience, healthy relationships, positive self-talk, realistic expectations, and self-respect, which are crucial for overall mental health and wellbeing, helping individuals navigate life’s challenges effectively.^16^

While a growing body of research links regular physical activity to improved physical and mental health compared with inactivity, there remains a gap in our understanding of how these factors relate to athletes at different sport performance levels, particularly regarding their self-esteem subjective health and wellbeing.^17–19^

The aim of this study is to investigate and compare the self-esteem, wellbeing, and health status among high-performance athletes, recreational athletes, and individuals with no or lower levels of physical activity aged 18–35 years. Another aim is to examine whether the type of sport (individual versus team) affects self-esteem and wellbeing based on sport performance.

## Methods

### Study design, participants, and recruitment

Quantitative, cross-sectional survey methodology was adopted for this observational study. Females aged 18–35 years were recruited in three different platforms. High-performance athletes were contacted through three sports clubs from two cities on voluntary nature. Recruitment was facilitated by coaches who helped establish contact with athletes. Control participants were selected from two tertiary education institutes from the same cities through the Student Electronic System, which facilitated widespread distribution of the survey invitation. Participants were also asked to share the link of the questionnaire with their female friends and relatives between the age of 18 and 35 years. Participants were categorized into three groups: High-performance athletes were considered those who registered with a sports club and actively competed in national-level competitions, either in the first or second division. Recreational athletes were defined as those who self-identified as such. This definition did not exclude individuals who were members of leisure sports clubs or who participated in recreational or mass sports events. Their weekly sport activity met the required 150 minutes of physical activity. Individuals with no or lower levels of physical activity were considered if they indicated that they did not engage in regular sports activities or if their weekly physical activity was less than 150 minutes.

### Ethics

Approval for the research was granted by the Regional/Institutional Scientific and Research Ethics Committee of the Borsod-Abaúj-Zemplén County Central Hospital and University Teaching Hospital under the number of BORS-15/2023. Participants were informed about the purpose and method of the research at the beginning of the questionnaire, and they were also informed that they would participate anonymously and voluntarily and could withdraw from the survey at any time. Written consent was given by ticking the appropriate box.

### Measures

#### Sociodemographic and sport-related variables

Participants were asked to provide information about their age, family status (living with parents, single, living in partnership, other form), occupation (studying at not tertiary education, studying at tertiary education, employed, involved only in sports performance), as well as duration of sport activity in years, weekly training hours, type of sport, and level of sport performance.

#### Self-esteem

The Rosenberg Self-Esteem Scale (RSES) is a widely used self-report instrument for evaluating individual self-esteem. Developed by Rosenberg (1965, 1989), it is considered one of the most reliable and valid measures of global self-esteem.^20,21^ The scale consists of 10 statements about self-worth and self-acceptance. Participants rate each statement on a 4-point Likert scale ranging from “strongly agree” to “strongly disagree.” Five of the items are positively worded, and five are negatively worded. In our research, we applied the linguistic validity and cultural adaptation of Sallay et al.^22^

#### Wellbeing

The WHO-5 Well-Being Index was used to measure the individual’s mental wellbeing. It is developed by the World Health Organization, and it focuses on positive mental health aspects. It consists of five positive statements about feelings experienced over the past two weeks. Participants respond to each statement using a 5-point Likert scale, ranging from “at no time” (0) to “all of the time” (5). To get raw data, the resulting sum was multiplied by 4. Higher scores indicate better wellbeing.^23^ The scale has adequate validity both as a screening tool for depression and as an outcome measure in clinical trials and has been applied successfully across a wide range of study fields. It has been validated for the Hungarian population by Susánszky et al.^24^

#### Health status

The EuroQol Visual Analogue Scale (VAS) records the patient’s self-rated health on a vertical visual analogue scale where the endpoints are labeled “0 = worst imaginable health state” and “100 = best imaginable health state.” The VAS can be used as a quantitative measure of health outcome that reflects the patient’s own judgement.^25,26^

### Data analysis

Statistical analysis was performed using IBM SPSS version 28.0 (Armonk, NY: IBM Corp.). Qualitative variables are presented as proportions and percentages, while quantitative variables are presented as means and standard deviations. Missing data were treated with mean substitution. A one-way analysis of variance (ANOVA) with Tukey *post hoc* test was used to determine the mean differences between the three investigated study groups (high-performance, recreational, and individuals with no or lower levels of physical activity) on self-esteem, wellbeing, and health. Before conducting the analysis, assumptions were checked. The dependent variables were measured at the interval level, the groups were independent of each other, Q–Q plots indicated normal distribution, and Levene’s test for homogeneity of variances was nonsignificant for all parameters. To analyze the difference in self-esteem and health between groups and assess whether it is affected by playing individual or team sports, a two-way ANOVA was used. The level of statistical significance was set at *p* ≤ 0.05.

## Results

### Participants

A total of 300 questionnaires were processed. The average age of the subjects was 21.95 years (SD = 3.53). Among the participants, 40.3% were high-performance athletes, 25.0% engaged in recreational sports, and 34.7% reported no or low levels of physical activity. Within the high-performance athlete group, 22% played football/soccer, 18% played volleyball, 14% played basketball, 12% participated in athletics, 7% played handball, and 3% engaged in road cycling and swimming. In addition, 2% were involved in wrestling, karate, fencing, cheerleading, horse riding, ice hockey, weightlifting, and gymnastics, whereas 1% participated in acrobatic rock and roll, figure skating, sport climbing, taekwondo, and water polo. Among recreational athletes, 17% chose one of the dance forms, 16% participated in conditioning exercises, 12% in athletics, 8% in basketball and horse riding, 7% in volleyball, gymnastics, and fitness classes, 4% in kayaking, swimming, and tennis, 3% in handball, and 1% in cycling, fencing, and ultimate frisbee. Table 1 displays characteristics of the participants based on their level of sport performance.

**Table 1.** Characteristics of Participants Based on Their Level of Sport Performance (*n* = 300)

Mean (standard deviation) proportions and percentages	High-performance athletes	Recreational athletes	No or lower levels of PA
Sample size	121	75	104
Age (years)	21.43 (3.08)	22.31 (3.86)	22.30 (3.72)
Family status (%)			
Living with parents	54.5	65.3	65.4
Single	19.8	8.0	5.8
Living in partnership	19.0	18.7	15.4
Other	6.6	6.7	11.5
Missing data	—	1.3	1.9
Occupation (%)			
Student at nontertiary education	11.6	5.3	6.7
Student at tertiary education	66.9	81.3	78.8
Employed	6.6	13.3	9.6
Only does sport	14.9	—	—
Other	—	—	1.0
Duration of sport activity (years)^a^	14.51 (4.41)^*^	12.04 (5.19)	—
Weekly training hours	13.52 (5.92)^*^	5.71 (2.98)	—
Type of sport (%)			
Individual sport	33.1	77.8	—
Team sport	66.9	22.2	—

Notes: Mean values are presented with standard deviations in parentheses where applicable.

“-” indicates data that is not applicable or missing.

Student *t*-test between high-performance and recreational athletes.

^a^ It should be mentioned there was a significant correlation between age and duration of sport activity (r = 0.766; *p* < 0.001).

^*^ *p* < 0.001.

### Self-esteem, wellbeing, and health status

Comparing self-esteem, wellbeing, and health status, significant differences were found among study groups. The high-performance athletes reported the most favorable scores in all parameters. (Table 2)

**Table 2.** Comparison of Self-Esteem, Wellbeing, and Health Status Among High-Performance, Recreational, and Nonathletes (*n* = 300)

Mean (standard deviation)	High-performance athletes	Recreational athletes	No or lower level of PA	F-distribution
Self-esteem	30.43 (5.22)	28.91 (5.83)	27.38 (6.20)	7.959^**^
Wellbeing	58.57 (19.21)	54.83 (19.04)	47.23 (20.07)	9.338^**^
Health status	81.78 (15.12)	78.19 (15.47)	73.60 (17.81)	6.885^*^

^*^ *p* = 0.001.

^**^ *p* < 0.001.

In the multiple comparison, Tukey post-hoc test revealed significant differences between high-performance athletes and individuals with no or low level of physical activity in all three parameters, and between recreational and individuals with no or low level of physical activity in wellbeing. (Table 3)

**Table 3.** Tukey Post-Hoc Test on Self-Esteem, Wellbeing, and Health Status Among Study Groups (*n* = 300)

	Mean differences	Standard error	*p* value
Self-esteem			
High-performance			
Recreational	1.523	0.842	0.168
No or lower level of PA	3.055	0.766	**<0.001**
Recreational			
High-performance	−1.523	0.842	0.168
No or lower level of PA	1.532	0.868	0.183
No or lower level of PA			
High-performance	−3.055	0.766	**<0.001**
Recreational	−1.532	0.868	0.183
Wellbeing			
High-performance			
Recreational	3.740	2.865	0.393
No or lower level of PA	11.334	2.643	**<0.001**
Recreational			
High-performance	−3.740	2.865	0.393
No or lower level of PA	7.594	2.980	**0.030**
No or lower level of PA			
High-performance	−11.334	2.643	**<0.001**
Recreational	−7.594	2.980	**0.030**
Health status			
High-performance			
Recreational	3.595	2.382	0.288
No or lower level of PA	8.179	2.204	**<0.001**
Recreational			
High-performance	−3.595	2.382	0.288
No or lower level of PA	4.585	2.479	0.156
No or lower level of PA			
High-performance	−8.179	2.204	**<0.001**
Recreational	−4.585	2.479	0.156

To examine whether the type of sport (individual vs. team) affects self-esteem among high-performance and recreational athletes, a two-way ANOVA was conducted. Descriptive statistics indicated that the mean self-esteem scores were 30.43 (SD = 5.22) for individual sports and 28.82 (SD = 5.91) for team sports. The analysis revealed no significant main effect of the type of sport on self-esteem, F = 1.961, *p* = 0.163, η^2^ = 0.010. This suggests that there is no statistically significant difference in self-esteem between athletes participating in individual sports compared to those in team sports. The interaction effect between the type of sport and the level of athleticism (high-performance vs. recreational) was also nonsignificant (F = 0.016, *p* = 0.900, η^2^ < 0.001).

Running the same analysis on wellbeing, similar results were observed. The wellbeing score for individual sport was 58.57 (SD = 19.21), for team sport 54.61 (SD = 19.26) without significant main effect of the type of sport (F = 0.122, *p* = 0.683, η^2^ = 0.001). The interaction effect between the type of sport and the level of athleticism was also nonsignificant (F = 2.402, *p* = 0.123, η^2^ = 0.013).

## Discussion

The present study aimed to investigate the differences in self-esteem, subjective wellbeing, and health status among athletes with varying levels of sports performance while also exploring the potential impact of individual versus team sports on these parameters. Our findings highlight variations across these groups, providing insights into the benefits of regular sport participation and the distinct experiences of athletes at different levels of engagement.

Based on the results, high-performance athletes demonstrated the highest levels of self-esteem, wellbeing, and subjective health status, suggesting that intense and competitive physical activity fosters a strong sense of self-worth and overall health. Some studies have suggested that the high physical and mental demands of high-performance sports can negatively affect athletes.^6,8,27^ In contrast, our study suggests that the commitment associated with high-performance sports may contribute to enhanced psychological wellbeing and physical health. The rigorous discipline, goal-oriented mindset, strong resilience, effective coping mechanisms, and social support inherent in high-performance sports might foster a positive self-image, self-esteem, overall life satisfaction, and strategies to handle competitive pressure. High-performance athletes are generally in peak physical condition, which positively influences their self-rated health status. This aligns with previous research that has found structured, high-level engagement in sports to be beneficial for mental health and wellbeing.^28–30^ It should be mentioned these athletes have high level of background assistants such as medical professions, sports psychologists, nutritionists, physiotherapists, or masseurs beyond the high-quality trainers, which positively contribute their health status. However, statistically significant differences were observed only when comparing high-performance athletes with the individuals with no or low level of physical activity. Recreational athletes did not show significant differences from either high-performance athletes or individuals with no or low level of physical activity, except in wellbeing.

Both high-performance and recreational sport performance contributes positively to the overall physical and mental health. The regular engagement in sports and exercise could lead to similar levels of self-esteem, wellbeing, and health status, minimizing the differences between these two groups. High-performance athletes may engage in higher intensity and more frequent training, but recreational athletes also maintain a level of physical activity that significantly benefits their wellbeing and health. The health benefits of regular physical activity may plateau at a certain point, beyond which additional activity (as seen in high-performance athletes) does not lead to significantly higher self-esteem, wellbeing or health status. While high-performance athletes face higher levels of competitive stress, they often have access to better support systems (*e.g.*, sports psychologists) to help manage this stress. Recreational athletes, on the other hand, engage in sports primarily for enjoyment and health, which might balance out the stress levels when compared to high-performance athletes.

Previous studies suggested team sport superior to individual sport;^31–33^ however, Sagat et al. found individual athletes presented a higher level of self-esteem.^34^ Interestingly, our study found no significant differences in self-esteem and wellbeing between participants in individual sports and those in team sports, which is in line with Eather et al.’s findings.^35^ This suggests that the type of sport might be less important than the act of regular physical engagement itself. Both individual and team sports require discipline, goal-setting, and physical effort, which are beneficial for psychological health. This finding emphasizes that promoting any form of physical activity can be effective in enhancing self-esteem and wellbeing, regardless of whether the activity is performed alone or in a group setting.

### Limitation and future directions

This study is not without its limitations. The cross-sectional design precludes any causal inferences, and the reliance on self-reported data may introduce response biases. Moreover, the sample may be biased toward individuals who continue to participate in sports because they find it enjoyable or beneficial. Those who might have experienced diminished self-esteem, poor wellbeing, or adverse health outcomes from sports participation may have dropped out and, as a result, are underrepresented in our study. This self-selection bias could have led to an overestimation of the positive effects of sports participation on wellbeing and self-esteem and an underrepresentation of potential negative outcomes. While the instruments used in the study are widely employed in the literature, they might not be sensitive enough to detect subtle differences between high-performance and recreational athletes. Future research should consider longitudinal designs to track changes in wellbeing and self-esteem over time and account for individuals who discontinue sports participation. In addition, studies could explore the reasons for dropping out of sports, which could provide a more comprehensive understanding of the full range of experiences related to athletic involvement. Future research with larger and more diverse samples could provide greater insight into the potential differences between individual and team sport athletes. In addition, qualitative research could explore the nuanced experiences of athletes in different sports, contributing to a deeper understanding of how sport type influences self-esteem and wellbeing.

## Conclusion

In conclusion, the findings from this study suggest that engaging in sports, whether at a recreational or high-performance level, is associated with mental and physical health benefits. While recreational athletes benefit from regular physical activity, high-performance athletes may experience additional psychological benefits from the structured and intensive nature of their training and competition. However, the lack of significant differences in self-esteem, wellbeing, and health status between high-performance and recreational athletes indicates that the benefits of physical activity are substantial at both levels.

It is important to acknowledge that these conclusions are drawn from a sample that may be biased toward individuals who continue participating in sports because they find it fulfilling or beneficial. As such, our study may not fully represent those who discontinued sports due to negative experiences. The higher stress and demands of high-performance sports may be offset by better support systems and coping mechanisms available to high-performance athletes, but this does not account for those who may have dropped out of organized sports altogether. Therefore, while our findings suggest that regular sports activity is generally beneficial for self-esteem, wellbeing, and health status, they should be interpreted with caution given the limitations of our study design.

Future research should further explore the experiences of those who leave sports, as well as the reasons for dropout, to provide a more comprehensive understanding of how different levels and types of sports participation affect psychological and physical outcomes. Nonetheless, the importance of maintaining a physically active lifestyle remains clear.

## Data Availability

The data that support the findings of this study are available from the corresponding author upon reasonable request.

## Abbreviations Used

ANOVA: one-way analysis of variance
PA: physical activity
RSES: Rosenberg Self-Esteem Scale
SD: standard deviation
VAS: visual analogue scale
vs: versus
WHO: World Health Organization

## References

[1] Khan KM, Thompson AM, Blair SN, et al. Sport and exercise as contributors to the health of nations. Lancet 2012;380(9836):59–64; doi: 10.1016/S0140-6736(12)60865-4 22770457

[2] Malm C, Jakobsson J, Isaksson A. Physical activity and sports-real health benefits: A review with insight into the public health of Sweden. Sports (Basel) 2019;7(5):127; doi: 10.3390/sports7050127 31126126 PMC6572041

[3] Posadzki P, Pieper D, Bajpai R, et al. Exercise/physical activity and health outcomes: An overview of Cochrane systematic reviews. BMC Public Health 2020;20(1):1724; doi: 10.1186/s12889-020-09855-3 33198717 PMC7670795

[4] Thyfault J, Bergouignan A. Exercise and metabolic health: Beyond skeletal muscle. Diabetologia 2020;63(8):1464–1474; doi: 10.1007/s00125-020-05177-6 32529412 PMC7377236

[5] Rice SM, Purcell R, De Silva S, et al. The mental health of elite athletes: A narrative systematic review. Sports Med 2016;46(9):1333–1353; doi: 10.1007/s40279-016-0492-2 26896951 PMC4996886

[6] Gustafsson H, Sagar SS, Stenling A. Fear of failure, psychological stress, and burnout among adolescent athletes competing in high level sport. Scand J Med Sci Sports 2017;27(12):2091–2102; doi: 10.1111/sms.12797 27882607

[7] Lundqvist C, Andersson G. Let’s talk about mental health and mental disorders in elite sports: A narrative review of theoretical perspectives. Front Psychol 2021;12:700829; doi: 10.3389/fpsyg.2021.700829 34267715 PMC8275956

[8] Walton CC, Purcell R, Henderson JL, et al. Mental health among elite youth athletes: A narrative overview to advance research and practice. Sports Health 2024;16(2):166–176; doi: 10.1177/19417381231219230 38173251 PMC10916785

[9] Meeusen R, Duclos M, Foster C, et al; European College of Sport Science, & American College of Sports Medicine. Prevention, diagnosis, and treatment of the overtraining syndrome: Joint consensus statement of the European College of Sport Science and the American College of Sports Medicine. Med Sci Sports Exerc 2013;45(1):186–205; doi: 10.1249/MSS.0b013e318279a10a 23247672

[10] Kreher JB, Schwartz JB. Overtraining syndrome: A practical guide. Sports Health 2012;4(2):128–138; doi: 10.1177/1941738111434406 23016079 PMC3435910

[11] Purcell R, Gwyther K, Rice SM. Mental health in elite athletes: Increased awareness requires an early intervention framework to respond to athlete needs. Sports Med - Open 2019;5(1):46; doi: 10.1186/s40798-019-0220-1 31781988 PMC6883009

[12] Bartholomew KJ, Ntoumanis N, Ryan RM, et al. Psychological need thwarting in the sport context: Assessing the darker side of athletic experience. J Sport Exerc Psychol 2011;33(1):75–102; doi: 10.1123/jsep.33.1.75 21451172

[13] Warburton DER, Bredin SSD. Health benefits of physical activity: A strengths-based approach. J Clin Med 2019;8(12):2044; doi: 10.3390/jcm8122044 31766502 PMC6947527

[14] Nikolaidis PT, Knechtle B, Quartiroli A. Editorial: Who runs? psychological, physiological and pathophysiological aspects of recreational endurance athletes. Front Psychol 2020;11:2247; doi: 10.3389/fpsyg.2020.02247 33013586 PMC7516278

[15] Zarotis G, Tokarski W. The effect of recreational sports on human health. IOSR J Sports Phys Educ 2020;7(3):25–33; doi: 10.9790/6737-07032533

[16] Branden N. The Power of Self-Esteem. Health Communications, Inc: Deerfield Beach, FL; 1992.

[17] Schuch FB, Vancampfort D, Firth J, et al. Physical activity and incident depression: A meta-analysis of prospective cohort studies. Am J Psychiatry 2018;175(7):631–648; doi: 10.1176/appi.ajp.2018.17111194 29690792

[18] Alamdarloo GH, Shojaee S, Asadmanesh E, et al. A comparison of psychological well-being in athlete and non-athlete women. BJHPA 2019;11(2):109–116; doi: 10.29359/BJHPA.11.2.11

[19] Bueno-Antequera J, Munguía-Izquierdo D. Physical inactivity, sedentarism, and low fitness: a worldwide pandemic for public health. In: Integrated Science of Global Epidemics. Integrated Science, Vol 14. (Rezaei, N. eds.) Springer: Cham; 2023; doi: 10.1007/978-3-031-17778-1_19

[20] Rosenberg M. Society and the Adolescent Self-Image. Princeton University Press; Princeton, NJ; 1965.

[21] Rosenberg M. Society and the Adolescent Self-Image. (Rev. eds.). Wesleyan University Press: Middeltown, CT; 1989.

[22] Sallay V, Martos T, Földvári M, et al. Hungarian version of the Rosenberg Self-esteem Scale (RSES-H): An alternative translation, structural invariance, and validity. Mentálhigiéné És Pszichoszomatika 2014;15(3):259–275; doi: 10.1556/mental.15.2014.3.7

[23] Topp CW, Østergaard SD, Søndergaard S, et al. The WHO-5 Well-Being Index: A systematic review of the literature. Psychother Psychosom 2015;84(3):167–176; doi: 10.1159/000376585 25831962

[24] Susánszky E, Konkoly Thege B, Stauder A, et al. Validation of the short (5-item) version of the who well-being scale based on a Hungarian representative health survey (Hungarostudy 2002). Metálhigiéné És Pszichoszomatika 2006;3:247–255; doi: 10.1556/Mental.7.2006.3.8

[25] Hayes MHS, Patterson DG. Experimental development of the graphic rating method. Psychol Bull 1921;18:98–99.

[26] EuroQol Group. EuroQol–a new facility for the measurement of health-related quality of life. Health Policy (Amsterdam, Netherlands) 1990;16(3):199–208; doi: 10.1016/0168-8510(90)90421-9 10109801

[27] Schaal K, Tafflet M, Nassif H, et al. Psychological balance in high level athletes: Gender-based differences and sport-specific patterns. PLoS One 2011;6(5):e19007; doi: 10.1371/journal.pone.0019007 21573222 PMC3087722

[28] Eime RM, Young JA, Harvey JT, et al. A systematic review of the psychological and social benefits of participation in sport for adults: Informing development of a conceptual model of health through sport. Int J Behav Nutr Phys Act 2013;10(1):135; doi: 10.1186/1479-5868-10-135 24313992 PMC4028858

[29] Guddal MH, Stensland SØ, Småstuen MC, et al. Physical activity and sport participation among adolescents: Associations with mental health in different age groups. Results from the Young-HUNT study: A cross-sectional survey. BMJ Open 2019;9(9):e028555; doi: 10.1136/bmjopen-2018-028555 PMC6731817 31488476

[30] Snedden TR, Scerpella J, Kliethermes SA, et al. Sport and physical activity level impacts health-related quality of life among collegiate students. Am J Health Promot 2019;33(5):675–682; doi: 10.1177/0890117118817715 30586999 PMC7213817

[31] Sabiston CM, Jewett R, Ashdown-Franks G, et al. Number of years of team and individual sport participation during adolescence and depressive symptoms in early adulthood. J Sport Exerc Psychol 2016;38(1):105–110; doi: 10.1123/jsep.2015-0175 27018562

[32] Andersen MH, Ottesen L, Thing LF. The social and psychological health outcomes of team sport participation in adults: An integrative review of research. Scand J Public Health 2019;47(8):832–850; doi: 10.1177/1403494818791405 30113260

[33] Pluhar E, McCracken C, Griffith KL, et al. Team sport athletes may be less likely to suffer anxiety or depression than individual sport athletes. J Sports Sci Med 2019;18(3):490–496. 31427871 PMC6683619

[34] Šagát P, Bartik P, Lazić A, et al. Self-esteem, individual versus team sports. Int J Environ Res Public Health 2021;18(24):12915; doi: 10.3390/ijerph182412915 34948525 PMC8701405

[35] Eather N, Wade L, Pankowiak A, et al. The impact of sports participation on mental health and social outcomes in adults: A systematic review and the ‘Mental Health through Sport’ conceptual model. Syst Rev 2023;12(1):102; doi: 10.1186/s13643-023-02264-8 37344901 PMC10286465

